# Posterior urethral valves: 10 years audit of epidemiologic, diagnostic and therapeutic aspects in Yaoundé gynaeco-obstetric and paediatric hospital

**DOI:** 10.1186/s12894-018-0364-1

**Published:** 2018-05-21

**Authors:** Faustin Felicien Mouafo Tambo, Paul Nkemtendong Tolefac, Marcelin Ngowe Ngowe, Jacqueline Ze Minkande, Landry Mbouche, Georgette Guemkam, Neville Alemnju Telelen, Fru Fobuzshi Angwafo, Aurelien Maurice Sosso

**Affiliations:** 1 Specialized Internship Program, Faculty of Medicine and Biomedical Sciences, University of Yaoundé 1, Yaoundé, Cameroon; 2 Department of Surgery, Faculty of Health Sciences, University of Buea, Buea, Cameroon; 3 Department of Anaesthesia and Intensive Care, Faculty of Medicine and Biomedical Sciences, University of Yaoundé 1, Yaoundé, Cameroon; 4 Department of Anaesthesia and Intensive Care, Yaoundé Gynaeco-Obstetric and Paediatric Hospital, Yaoundé, Cameroon; 5 Paediatric Nephrology Unit, Mother and Child Centre, Chantal Biya Foundation, Yaoundé, Cameroon; 6 Departmennt of Surgery and Subspecialties, Faculty of Medicine and Biomedical Sciences, University of Yaoundé 1, Yaoundé, Cameroon; 7 Paediatric Surgery Unit, Yaoundé Gynaeco-Obstetric and Paediatric Hospital, Yaoundé, Cameroon

**Keywords:** Posterior urethral valves, Epidemiologic, Diagnostic, Yaoundé, Late presentation

## Abstract

**Background:**

The incidence of posterior urethral valve (PUV) is estimated at 1:5000–1:8000 males. It is the most common paediatric urologic urgency and the most common cause of male obstructive uropathy and chronic renal failure in children. The study aimed to describe the experience of Yaoundé gynaeco-obstetrics and paediatric hospital in the management of PUV.

**Methods:**

Retrospectively, medical records were retrieved over a ten year period and all data recorded and analyzed for study objectives. Patients were called and evaluated for outcomes regarding morbidity and mortality.

**Results:**

A total of 18 patients all males were managed over the ten year period, given prevalence of 13 cases/100,000 admissions and an admission rate of 2 per annum. The median age at presentation was 22 months and 13 (72.2%) participants presented late. Voiding urethrocystogram was done in all the participants where it showed dilated and elongated posterior urethral valves in 16 (88.9%) of the cases. Endoscopic valve ablation resulted in the relief of obstruction in all but 3 (16.7%) participants that had residual valves and 2 (11.2%) participants that had urethral stenosis. Type I valves were most common in 14 (78.0%) participants. The mean duration of follow up was 34.56 ± 21.47 months. Complications at final follow up were: 10 (55.6%) chronic renal failure, 2 (11.2%) end-stage renal failure. The case fatality rate was 5.6%.

**Conclusion:**

Many patients present late in our setting with already established complications. There is the need to counsel parents/guardians on the importance of long-term follow up after relief of obstruction.

## Background

Posterior urethral valve (PUV) is the most common lower urinary tract congenital anomaly in boys with an incidence of 1: 5000–1:8000 males [1–5]. It is the leading cause of bladder outlet obstruction and renal insufficiency in male children [6–8]. The incidence is unknown in our sub-region. In Nigeria, Uba et al. [3] in Jos, reported 3–8 cases per annum and Jaja et al. [9] in Port Harcourt observed that it accounted for 1 in 2447 children seen in their hospital.

This disease was first described by Morgagni in 1717 and later by Langenbeck in 1802 [10, 11]. The exact embryology and aetiology of this condition are not known.

PUV has detrimental and wide-ranging effects on the global development of the kidney, bladder and the entire urinary system. This result from persistent and unrelieved pressure on the bladder leading to bladder diverticular, hydronephrosis and chronic to end-stage renal diseases (ESRD). The anomaly is associated with high mortality and morbidity including urosepsis, overflow urinary incontinence, chronic kidney diseases (CKD), hypertension, chronic anaemia, failure to thrive, poor quality of life and even death [5, 12].

Early diagnosis can be made prenatally by regular and routine prenatal ultrasounds. The routine use of this modality has significantly increased early diagnosis and management of this pathology in most developed countries. As prenatal hydronephrosis usually prompts an early and complete work up at birth with a voiding cysto-urethrogram (VCUG) leading to early postnatal diagnosis [4]. Prenatal ultrasound uptakes are still at low levels in our environment.

Initial continuous catheter drainage and or vesicostomy and eventual management by endoscopic valve resection, usually ameliorate the obstructive uropathy. However, it has been noted that even after surgical treatment of valves and relief of obstruction, about 70% of older children and adolescents continue to have persistent bladder dysfunction, poor quality of life, and worsening CKD and ESRD [4, 11]. Long-term management of PUV constitutes a challenge in the practice of pediatric urology in our environment due to lack of specialized centers as well as the paucity of trained pediatric urologists and nephrologists to evaluate and monitor these children [3]. In the few highly specialized centers where valve ablation is done, most patients fail to turn up for follow-up visits due to an erroneous belief that it has been cured following the initial relief of the obstruction.

This study aimed to describe the epidemiologic, diagnostic and therapeutic aspects of PUV in Yaoundé Gynaeco-Obstetric and Paediatric Hospital (YGOPH), Cameroon. YGOPH is a tertiary care hospital located in the city of Yaoundé with a modern theatre that operates among other cases patients with PUV. These patients with PUV are usually operated in this center by a group of surgeons from China and Cameroon owing to the Cameroon-China cooperation.

## Methods

### Study design and setting

This was a retrospective descriptive cross-sectional study carried out over a period of 10 years (April 2005–March 2016) in the pediatric surgical unit of Yaoundé gynaeco-obstetric and pediatric hospital (YGOPH). YGOPH is a tertiary hospital located in the city of Yaoundé. Among other units in the hospital is the pediatric surgical unit that manages cases of pediatric surgery. It receives cases from Yaoundé and its environs. Cases of posterior urethral valves are usually received in the service all year round. The pediatric surgery unit is fully equipped material resources including a hospitalization section (12 beds), outpatient surgical consultations, minor theatre and the major theatre as well as human resources such as two pediatric surgeons, two urologists, one orthopedic surgeon and several nurses. Over the past years, corporation between this hospital and China has allowed these children to benefit from endoscopic resection of the valve by electrocautery. At hospitalization and after fluid and electrolyte stabilization, vesicostomy is usually done to relieve obstruction after which an interval endoscopic valve resection is programmed. Endoscopic valve resection is usually done by a team of Cameroonian and Chinese surgeons owing to the Cameroon-China cooperation in this hospital.

### Data collection

We included all cases of posterior urethral valves that had a confirmed endoscopic diagnosis, and we excluded all those whose medical files could not be seen as well as those with files not containing relevant information such as endoscopic findings and valve types. Those with alternative endoscopic intraoperative diagnosis were also excluded. The study was divided into two phases: Phase I was a was a retrospective analysis of children aged 15 years and below managed for PUV at the paediatric surgical unit of YGOPH for 10 years between April 2005 to March 2016. Phase II was a cross-sectional evaluation of the clinical and kidney functions of all the patients operated between the said period. Information on the age at presentation, clinical features, and anthropometric measures, results of investigations: hemoglobin level, serum electrolytes, white cell count, urea and creatinine, urine culture as well as information on prenatal ultrasound, postnatal abdominal ultrasound, voiding cysto-urethrogram (VCUG) were obtained. At each follow-up, weight, height, blood pressure, serum creatinine, and full blood count and urine culture, complications developed were noted. After stabilizing the patients by controlling infection, correction of fluid, electrolyte balance, catheter drainage and or vesicostomy was done while awaiting definitive valve ablation which was programmed by a group of surgeons. After valve ablation, a urethral catheter was left in situ for 3–5 days to allow the oedema to subside and to enable measurement of urinary output. Intravenous fluids were given and adjusted to meet the need of patients while serum electrolytes were monitored. Patients were then subsequently discharged on antibiotic prophylaxis and followed up in an outpatient clinic at pediatric nephrology consultation at the mother and child center, Chantal Biya Foundation with voiding history, abdominopelvic ultrasonography, and serial serum creatinine estimation.

### Statistical analysis

Data collected with the aid of case report forms were entered into WHO epi info version 3.5.4 and analyzed using WHO epi info version 3.5.4 and statistical package for social scientists (SPSS) software version 20. The results were presented as tables and charts. Mean and standard deviation were determined for continuous variables and frequency and percentage for categorical variables. Continuous variables were compared with t-test, and categorical variables with Chi-Square test or the McNemar test were appropriate. Level of significance was set at a *p*-value 0f < 0.05.

### Ethical considerations

Ethical clearance was obtained from the ethical committee of YGOPH and the institutional review board of the University of Douala.

### Definition of terms

Late presentation is defined in this study as a patient presenting after 30 days of life.

## Results

### Sociodemographic and clinical characteristics of posterior urethral valves

There were 140,444 admissions of ages 0 days to 15 years patients in the pediatric surgery unit of YGOPH between April 2005 and March 2016, out of which 25 were treated for posterior urethral valves (PUV). According to hospital records 25 patients were hospitalized for PUV during the study period, 7 participants were excluded, in five of them the medical files were missing, and in the remaining two the files were incomplete with missing vital information such as endoscopic findings, types of valves and VCUG findings (Table 1).

**Table 1:** Age distribution at presentation

Age group / months	Frequency (n)	Percentage (%)
≤1 month	5	27.8
2-12 months	3	16.7
13-48 months	4	22.2
49-84 months	4	22.2
85-120 months	2	11.1
Total	18	100

Their age at presentation ranges from 1st day of life to 108 months (9 years) with a median age of 22 months. The diagnosis was made in 13 (72.2%) participants after 1 month and in 10 (55.6%) after 1 year as shown in Table 1. This gives a prevalence of 13 cases of PUV/100, 000 admissions in our pediatric surgery unit and an annual admission rate of 2 cases of PUV per annum

**Fig. 1:**
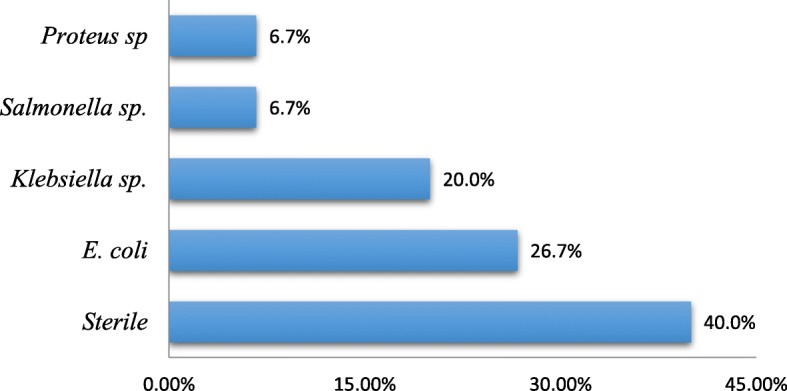
Bacteria isolated in the urine of patients

As shown in Table 2, the most common clinical features were voiding dysfunction (characterized by dribbling, poor stream, and straining) in all 18 (100%) participants, supra-pubic distension from palpable bladder in 9 (50.0%) participants. Others included recurrent UTI in 11 (61.1%) participants, anaemia in 10 (55.6%) participants, urine retention with suprapubic bladder in 9 (50.0%) participants, fever with core temperature > 38 °C in 7 (38.9%) participants, failure to thrive in 5 (27.8%) participants, ascites in 4 (22.4%) participants; associated congenital malformations noted were umbilical hernia in 4 (22.2%) participants, cryptorchidism in 2 (11.1) participants, vaginal hydrocele in 1 (5.6%) participants and inguinal hernia 1 (5.6%) participants, 15 (83.3%) did urine culture at presentation, 6 (40.0%) of the urine culture were sterile. The most frequently isolated bacteria were *Escherichia coli* in 4 (26.7%) and *Klebsiella sp.* in 3 (20.0%). The bar chart in Fig. 1 that follows is a distribution of the bacteria isolated.

**Table 2:** Clinical features at presentation

Clinical Feature	Frequency (n)	Percentage (%)
Voiding dysfunctions	18	100
Fever	7	38.9
Convulsion	2	11.1
Gastroenteritis	2	11.1
Respiratory Distress	2	11.1
Sepsis	2	11.1
CKD	2	11.1
Recurrent UTI	11	61.1
Failure to thrive	5	27.8
History Neonatal infection	4	22.2
Jaundice	2	11.1
Lower limb oedema	1	5.6
Abdominal Distension/Ascites	4	22.2
Urine retention	9	50.0
Umbilical hernia	4	22.2
Inguinal hernia	1	5.6
Vaginal hydrocele	1	5.6
Cryptorchidism	2	11.1
Anaemia	10	55.6

### Diagnosis of posterior urethral valves

**Fig. 2:**
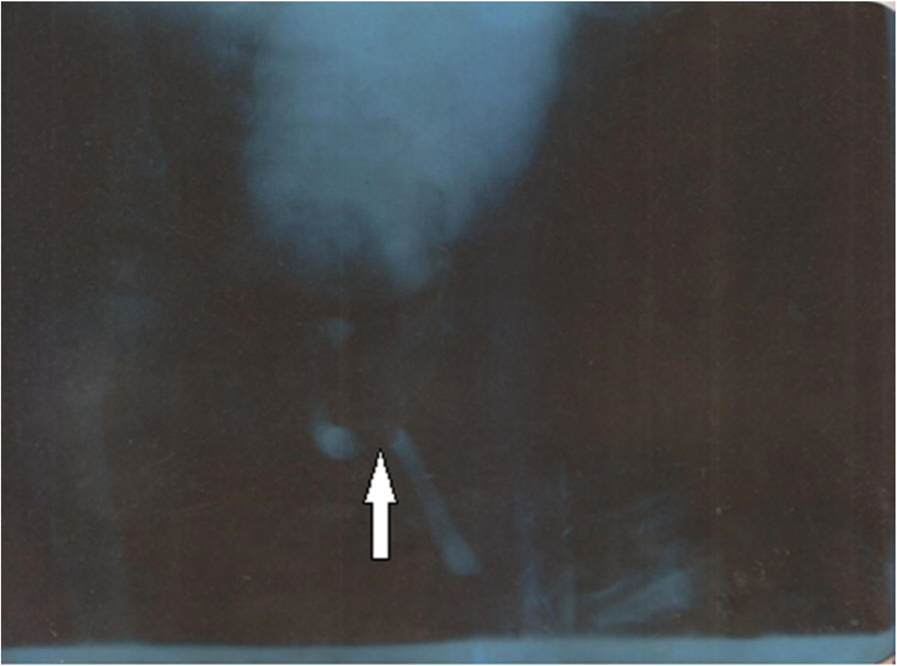
VCUG showing dilated and elongated PUV (White arrow) in a 2 year 3 months old toddler

VCUG was diagnostic in 16 (88.9%) patients, showing dilated and elongated posterior urethra. Figure 2 is a VCUG of a 2.3 year-old toddler showing dilated and elongated PUV (white arrow). There was associated vesico-ureteric reflux in 10 (55.6%) patients; bladder diverticular in 3 (16.7%) and multiple trabeculated bladder in 8 (44.4%). Abdominal Ultrasound was diagnostic in 7 (38.9%) showing dilated and elongated posterior urethral valves. In 16 (88.9%) participants there was suspicion of PUV by the presence of bilateral hydronephrosis (BHN). Out of the participants with BHN, 6 (37.5%) had stage I BHN, 6 (37.5%) had stage II BHN, 2 (12.5%) had stage III BHN and 2 (12.5%) had stage IV BHN. Other ultrasound findings included vesico-ureteric reflux 9 (50.0%), trabeculated bladder 11 (61.1%). 10 (55.6%) participants did a prenatal ultrasound between 18th to 28th weeks of pregnancy. 2 (20%) of the prenatal ultrasounds were suggestive of PUV by showing bilateral hydronephrosis.

### Management of posterior urethral valves

All participants benefited from primary endoscopic valve resection by electrocautery. In 6 (33.3%) participants vesicostomy for urinary drainage was done before definitive endoscopic valve resection. Valve resection resulted in improvement of urinary symptoms in all but (3) 16.7% of participants that had residual valves that necessitated a second endoscopic resection and in 2 (11.2%) participants it was complicated by urethral stenosis that necessitated urethral dilatation. The delay between diagnosis and primary endoscopic valve resection ranges between 0 and 46 months (median = 1 month) whereas the mean delay between vesicostomy and urethrocystoscopy ranges between 0 and 12 months. The most frequent valves were type I in 14 (77.8%) participants. Type II and type III valves had an equal prevalence of 11.2% each.

### Complications of posterior urethral valves

**Fig. 3:**
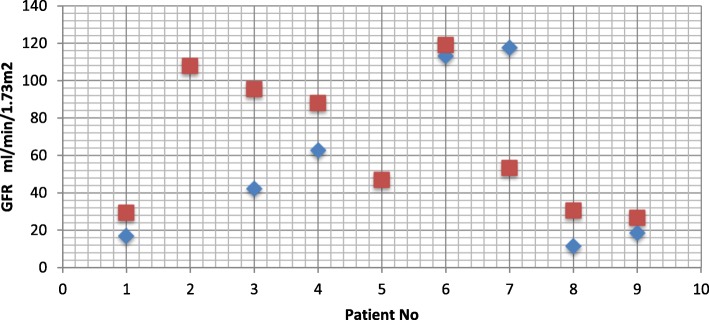
Variations of GFR before surgery (blue) and at final follow up (red) for 9 patients

At presentation, 6 (33.3%) patients were in renal insufficiency with serum creatinine greater than 132.6 μmol/l. However, determination of glomerular filtration rate (GFR) using Swartz formula showed a mean GFR of 51.0 ± 43.83 ml/min/1.73m^2^ with a range of 11.40–148.5 ml/min/1.73m^2^ and most of the participants having a GFR between 15 and 29 ml/min/1.73m^2^. Half of the participants were in chronic renal failure (GFR < 60) and16.70% in ESRD (GFR < 15) requiring dialysis. GFR could not be gotten for three participants with serum creatinine because their heights were not taking on admission. For the nine participants followed up (prospective phase), at diagnosis of posterior urethral valves, 4 (44.4%) patients were in chronic renal failure (GFR < 60) and 1 (11.1%) patients in ESRD (GFR < 15). At the final follow up, 5 (55.6%) participants were in CRF (GFR < 60) with 1 (11.1%) participants in ESRD (GFR < 15). The change in the percentage of patients with CRF before surgery and at follow up was not statistically significant using the McNemar test with the level of significance set at *p*-value of 0.05 (p-value =1). In general, as shown on the curve below (Fig. 3), there is a modest improvement of GFR and hence renal functions following relief of the obstruction.

The mean duration of follow up was 34.6 ± 21.5 months with a range of 6–73. 9 (50%) participants were lost to follow up. One death was recorded in 2013 with the cause of death being ESRF and urosepsis. This gives a mortality/case fatality rate (CFR) of 5.6%.

## Discussion

The indexed study described the experience in the management of posterior urethral valves in a tertiary care center in Cameroon. The prevalence of PUV and admission rate in the surgical unit of this institution was estimated at 13 cases / 100,000 admissions and 2 cases per annum respectively. An earlier review of childhood obstructive uropathy in Nigeria by Jaja et al. [9] in 2012 suggested that the condition is certainly not as rare as the paucity of reports on the prevalence in English literature may suggest [3]. In the present study, the rate of two children with PUV diagnosed yearly is comparable to earlier studies conducted in Cameroon by Angwafo et al. [13] and Chiabi et al. [14] where the rate ranges between 3 and 4 cases per annum. From this, we can conclude that this disease has a constant rate of presentation in our hospitals. This was equally similar to studies conducted elsewhere in Africa by Talabi et al. [2] where they got 3 per annum and Uba et al. [3] of 3–8 per annum. The low prevalence in our environment may be related to early death in the perinatal and neonatal periods as a result of severe obstruction, superimposed infection, acute renal failure and pulmonary hypoplasia, who never get to the hospital and were therefore never diagnosed [3, 15].

While the diagnosis of posterior urethral valves can be made prenatally [16, 17], only 10 (55.6%) patients did a prenatal ultrasound. In 2 (11.2%) of the ultrasounds, there was suspicion of PUV. This is consistent with reports by Okafor et al. [18] and Talabi et al. [2] of 6.5 and 8.1% respectively. A study conducted in Nigeria in 2010 by Ohagwu et al. [19] showed that negative attitude, long distances to service providers, considerably heavy financial cost, long waiting periods, and unsatisfactory previous scan experience are major barriers to prenatal ultrasound in Nigeria, and these barriers have indirectly made prenatal diagnosis of PUV difficult in our setting. Low level of detection of PUV by prenatal ultrasounds can also be explained by the lack of experts in prenatal imaging hence missed diagnosis.

The median age of diagnosis in the study of 22 months suggested that patients with this condition presented late in our environment. 72.7% presented after the age of 1 month and 55.5% presented after the age of 1 year. This is similar to studies done earlier in Cameroon by Chiabi et al. [14] where 50% of patients presented after 1 year. It is also similar to studies conducted in Nigeria by Orumah et al. [6], Odetunde et al. [1] and Ikuerowo et al. [20] where they found out that 56.8, 52 and 57.1% of patients respectively presented after the age of 1 year. Factors that might have contributed to late presentation noted include a suboptimal prenatal ultrasound utilization, [19] poverty, ignorance on the part of parents, the paucity of specialist care, late referral by primary care providers.

All the participants presented with voiding dysfunction ranging from dribbling 11 (61.1%), dysuria 8 (44.4%) and **pollakiuria** 6 (33.3%). This is similar to the study conducted earlier in Cameroon by Chiabi et al. [14] where the most common urinary symptoms were dribbling (61.7%) and dysuria (54%) and in another study by India by Bhaumik et al. [21] where 79% presented with dribbling. Again, the similarity was noted in studies conducted in Nigeria by Odetunde et al. [1] and Orumuah et al. [6] where 100 and 92% respectively presented with voiding anomaly. It is therefore important to have a high index of suspicion of PUV in neonates and infants presenting with urinary symptoms. We also found out that at hospitalization, nine (50.0%) of the participants had a palpable bladder/urine retention, which is comparable to a study conducted in a Nigerian tertiary hospital in 2015 by Orumuah et al. [6] where about a third of participants had a palpable bladder.

In our setting, participants were not routinely subjected to screening tests to detect other congenital anomalies due to financial constraints and the limited availability of screening facilities. However, associated congenital anomalies noted included 4 umbilical hernias (22.2%), one inguinal hernia (5.6%), one hydrocele (5.6%), and two cryptorchidism (11.2%). Similar congenital malformations were described in studies by Chiabi et al. [14] and Orumuah et al. [6]. Previous studies have also noted congenital defects in the cardiovascular, urogenital, gastrointestinal, and central nervous systems of males with PUV [6, 22, 23].

The gold standard for the diagnosis of PUV is VCUG [21]. This was done in all cases and demonstrated dilatation and elongation of the posterior urethral in (16) 88.9%, and can be compared to earlier studies in 2015 by Talabi et al. [2] where it showed dilated and elongated posterior urethral valves in 83.8% of the cases.

Biological investigations conducted at presentation revealed complications such as renal insufficiency, urinary tract infections, and anemia and electrolyte imbalances. (10) 55.6% patients had altered renal functions with glomerular filtration rate < 60 ml/min/1.73m^2^ at presentation which is comparable with 40.5% reported by Talabi et al. [2]. 71.4% reported by Odetunde et al. [1] in Nigeria, but in conflict with 15.4% reported by Dahab et al. [24]. Elevated serum creatinine level has been documented to be associated with poor prognosis in PUV patients, and this may not be reversed even with the relief of the obstruction. 60% of those who did urine cultures had a positive culture with the most commonly cultured germs being *Escherichia coli*. This was different from Talabi et al. [2] where the most common cultured bacteria was *Klebsiella sp*. These complications were similar to those observed in Nigeria [1, 6] and in Yaoundé [14].

Various operative techniques have been described for PUV resection. In this study, all the patients benefited from endoscopic valve resection. Type I valves were most common in 14 (78.0%) participants, which is comparable to what was found in a study in Nigeria [6]. Valve resection resulted in the relief of obstruction in all but three (16.7%) patients that had residual valves and thus needed repeat valve ablation to attain satisfactory passage of urine. This was similar to a study conducted by Mirshermirani et al. [12] and Sudarsanan et al. [25] where 15.3 and 13% respectively had residual valves. Endoscopic valve resection resulted to urethral stricture in two (11.2%) patients. This is comparable to 7.1, 5 and 8.2% obtained by Mirshermirani et al. [12], Shittu et al. [26] and Sudersanan et al. [25] respectively. Hence, the rate of complication in our low-resource setting remains relatively low and is comparable to that in the developed world which generally have greater resources and medical expertise. This comparison could be explained by the fact that the surgeries done in our setting are conducted by a team of both Cameroonian and Chinese surgeons thanks to the longstanding Cameroon-China cooperation.

The mortality rate of 5.6% recorded in our study is comparable to 4.9% reported in Nigeria [3], 5.1% in Iran [12] and 3.8% in Canada [27], but higher mortality rates were observed in Cameroon (21%) [14] in earlier studies conducted in Nigeria (9–13.5%) [2, 6, 18]. Studies have been reported with zero mortality rate [25] and other studies reported with mortality as high as 69% [1]. The reinforcement of our local team with surgeons from China, may explain the relatively low mortality in our series which is similar to that in the developed world.

The majority of the patients were lost to follow-up. Unfortunately, the reason for this is not known. However, it may be due to the reluctance of parents to continue hospital visits in the presence of satisfactory urine stream after relief of the obstruction. This, combined with the long distances required to travel to the hospital and poverty may also contribute to losses a follow-up. Educating parents and guardians about the importance of long-term follow-up and issues such as ongoing renal deterioration even after relief of obstruction should, therefore, be incorporated into the management of this disease.

### Limitations

Though our study has described very important clinico-biological characteristics of PUV in a major pediatric surgery unit in Cameroon, the applicability may be limited by:

- Cross-sectional nature and inability to establish spatial relationships
- A relatively small sample size of 18 participants
- Being solely hospital-based since many patients with PUV die during the neonatal period and infancy without an established diagnosis.
- Incomplete and absent information on many parameters as a result of its retrospective nature.

However, the study provides a documented perspectives of PUV over 10 years, providing much-needed evidence in a setting where the availability of data is a challenge. We, therefore, recommend a large multicentre case-control or cohort study to assess the relationship of most of the variables associated with PUV. In spite of these limitations, our study may serve as a pilot study for future studies on PUV and may provide data on clinical decision making in our local context.

## Conclusion

The rarity of posterior urethral valves with a rate of two cases per year, routine prenatal ultrasound and investigations of neonates presenting with urinary symptoms will lead to earlier detection. With early presentation, diagnosis, and, therefore, treatment, the outcome is expected to improve as valve ablation leads to satisfactory urinary stream in most of the patients in the series. The rate of complications and mortality remains relatively low. However, there is a need to further educate parents on the symptoms of the disease and the benefits of long-term follow–up of patients.

## Abbreviations

BHN: Bilateral hydronephrosis
CKD: Chronic kidney disease
CRF: Chronic renal failure
ESRD: End stage renal disease
GFR: Glomerular filtration rate
PUV: Posterior urethral valves
VCUG: Voiding cysto-urethrogram
YGOPH: Yaoundé Gynaeco – Obstetric and Paediatric Hospital

## References

[1] Odetunde OI, Odetunde OA, Ademuyiwa AO, Okafor HU, Ekwochi U, Azubuike JC (2012). Outcome of late presentation of posterior urethral valves in a resource-limited economy: challenges in management. Int J Nephrol.

[2] Talabi AO, Sowande OA, Etonyeaku AC, Salako AA, Adejuyigbe O (2015). Posterior urethral valves in children: pattern of presentation and outcome of initial treatment in Ile-Ife, Nigeria. Niger J Surg Off Publ Niger Surg Res Soc.

[3] Uba AF, Chirdan LB, Ihezue CH, Ramyil VL, Dakum NK (2007). Posterior urethral valves in children: pattern of presentation and outcome of initial treatment in Ile-Ife, Nigeria. Afr J Urol.

[4] Thomas J (2010). Etiopathogenesis and management of bladder dysfunction in patients with posterior urethral valves. Indian J Urol IJU J Urol Soc India.

[5] Desai D (2007). A review of urodynamic evaluation in children and its role in the management of boys with posterior urethral valves. Indian J Urol.

[6] Orumuah AJ, Oduagbon OE (2015). Presentation, management, and outcome of posterior urethral valves in a Nigerian tertiary hospital. Afr J Paediatr Surg.

[7] Hodges SJ, Patel B, McLorie G, Atala A (2009). Posterior urethral valves. ScientificWorldJournal.

[8] Uthup S, Binitha R, Geetha S, Hema R, Kailas L (2010). A follow-up study of children with posterior urethral valve. Indian J Nephrol.

[9] Jaja T, Anochie IC, Eke FU (2012). Posterior urethral valve in childhood in Port Harcourt, Nigeria. Port Harcourt Med J.

[10] Morgagni G, Alexander B (1769). The seats and causes of diseases investigated by anatomy; in five books, containing a great variety of dissections, with remarks. To which are added ... copious indexes.

[11] Nasir AA, Ameh EA, Abdur-Rahman LO, Adeniran JO, Abraham MK (2011). Posterior urethral valve. World J Pediatr.

[12] Mirshemirani A, Khaleghnejad A, Rouzrokh M, Sadeghi A, Mohajerzadeh L, Sharifian M (2013). Posterior urethral valves; a single center experience. Iran J Pediatr.

[13] Angwafo F, Andze G, Biouele JM, Sosso MA, Edzoa T, Niat G (1995). Les valves de l’urètre postérieur chez l’enfant : à propos de 22 cas. J Urol (Paris).

[14] Chiabi A, Angwafo F, Obama MT, Takou V, Zoung JK. Posterior urethral valves in children: a review of 28 cases in Yaounde, Cameroon. Clin Mother Child Heal. 2006;1(2) [cited 2016 Jan 18]. Available from: http://www.ajol.info/index.php/cmch/article/view/35799

[15] Tolefac PN, Tamambang RF, Yeika E, Mbwagbaw LT, Egbe TO (2017). Ten years analysis of stillbirth in a tertiary hospital in sub-Sahara Africa: a case control study. BMC Res Notes.

[16] Ka H, Df T, Rj A, Hc I, Se S (1994). Prenatally detected posterior urethral valves: is gestational age at detection a predictor of outcome?. J Urol.

[17] ACOG Practice Bulletin No. 101: Ultrasonography in pregnancy. - PubMed - NCBI [Internet]. [cited 2016 Apr 11]. Available from: http://www.ncbi.nlm.nih.gov/pubmed/19155920

[18] Okafor HU, Ekenze SO, Uwaezuoke SN (2013). Posterior urethral valves: determinants of outcome in a developing country: posterior urethral valve outcome. J Paediatr Child Health.

[19] Ohagwu CC, Abu PO, Odo MC, Chiegwu HU. Maternal perception of barriers to utilization of prenatal ultrasound in prenatal care in the northern part of Nigeria. Clin Mother Child Heal. 2010;7(1) [cited 2016 Apr 10] Available from: http://www.ajol.info/index.php/cmch/article/view/60569

[20] Ikuerowo SO, Balogun BO, Akintomide TE, Ikuerowo AOA, Akinola RA, Gbelee HO (2008). Clinical and radiological characteristics of Nigerian boys with posterior urethral valves. Pediatr Surg Int.

[21] Bhaumik K, Chatterjee I, Basu KS, Samanta N, Das S (2003). Posterior urethral valve : its clinical, biochemical and imaging patterns. J Indian Assoc Pediatr Surg.

[22] Heikkila J (2008). Posterior urethral valves are often associated with cryptorchidism and inguinal hernias. J Urol.

[23] Mondal K, Maheshwari A, Aneja S, Seth A (2012). A case of Down syndrome with a posterior urethral valve. Indian J Nephrol..

[24] Dahab H, Khalafallah AH, Mosaab. Posterior urethral valve presentation, management and outcome. Glob J Med Res. 2013;13(5) [cited 2016 Apr 18] Available from: http://medicalresearchjournal.org/index.php/GJMR/article/view/406.

[25] Sudarsanan B, Nasir AA, Puzhankara R, Kedari PM, Unnithan GR, Damisetti KRP (2009). Posterior urethral valves: a single center experience over 7 years. Pediatr Surg Int.

[26] Shittu OB, Asinobi AO (2004). Long-term outcome of posterior urethral valves ablation using the Mohan’s urethral valvotome. West Afr J Med.

[27] Warren J, Pike J, Leonard M (2004). Posterior urethral valves in Eastern Ontario - a 30 year perspective. Can J Urol.

